# Assessment of Health Literacy and Health Promoting Behaviors among the Urban Adult Population

**DOI:** 10.4314/ejhs.v32i5.14

**Published:** 2022-09

**Authors:** Soheila Ranjbaran, Khalil Maleki Chollou, Towhid Babazadeh

**Affiliations:** 1 Department of Public Health, Sarab Faculty of Medical Sciences, Sarab, Iran; 2 Department of Nursing, Sarab Faculty of Medical Sciences, Sarab, Iran; Department of Public Health, Sarab Faculty of Medical Sciences, Sarab, Iran

**Keywords:** Health Literacy, Health Promoting Behaviors, Adults, Iran

## Abstract

**Background:**

The main criteria of health determinants are Health Promoting Behaviors (HPBs) and Health Literacy (HL). This study aimed to assess HL and HPBs among the urban population of adults.

**Methods:**

This study was conducted as a cross-sectional study with 379 adults in Iran. The inclusion criteria was that participants were randomly selected from health centers using health records. Data were collected by demographic information questionnaire, Health Literacy for Iranian Adults (HELIA) and Health Promoting Lifestyle Profile II (HPLP-II). Descriptive statistics, independent samples t-test, and Pearson's r correlation coefficient were used to analyze the data. Also, the hierarchical regression analysis was used to adjust for confounders.

**Results:**

There was a significant association between HL and demographic factors, including age, gender and education level. HL dimensions were significantly correlated with HPBs of adults (p <0.05). Decision-making was the strongest HL dimension to predictive HPBs (β=0.606). In this study, 49.7% of the variation in HPBs is predicted by the HL, and the demographic characteristics.

**Conclusions:**

It can be advantageous to design programs to promote HL and HPBs in adults, focusing on the aging group, gender, and education level.

## Introduction

According to WHO's definition, “Health literacy represents the cognitive and social skills which determine the motivation and ability of individuals to gain access to, understand, and use the information in ways which promote and maintain good health” (1). A sufficient level of HL in individual leads to the ability to take responsibility for one's own health and one's family health and community health (2). A study conducted by Haghdoost et al. in Iran showed that 18% of the general adults population had sufficient HL, 45.7% had insufficient, and the 36.3% had moderate HL (3). In another study conducted in Turkey, 80.7% of the adults had low levels of HL (4).

Low HL is related to increased hospitalization rates, use of emergency services, problems in taking medications and understanding labels and health messages, poorer general health status, higher mortality rates, and decreased use of mammography screenings and influenza vaccinations (5). Also, smoking and alcohol abuse and low physical activity are behaviors related to poor levels of HL (6–9). Poor HL caused some consequences, including poor health self-assessment, raising obesity and disability, decreased physical activity, fruit and vegetable consumption, and more hospitalizations (9).

One of the main criteria of health determinants is HPBs (10), and according to a previous study, HL is significantly associated with HPBs (11,12). Rudd, Kirsch, and Yamamoto interpret health behaviors that are related to HL: “The range encompasses activities related to health promotion (e.g., purchase food), health protection (e.g., decide among product options and use products), disease prevention (e.g., undergo screening or diagnostic tests), health care and maintenance (e.g., calculate timing for medicine), and system navigation (e.g., locate facilities or apply for benefits)” (13).

High levels of HL in individuals lead to better health outcomes compared with people with low levels of HL (14). The results of a study in Iran revealed that increasing the HL led to improvements in HPBs in the health ambassadors (10), because people with higher levels of HL also have a better health perception (4). Low levels of HL result in low adherence to HPBs and a healthy lifestyle (15).

Due to the importance of HL and HPBs in adult populations, the lack of cohesive studies on both in Northwestern Iran, and the fact that age is associted to low levels of HL and HPBs (4, 11, 16, 17), we assessed HL and HPBs among the urban adult population.

## Methods

**Study design and participants**: This study was conducted as a cross-sectional study in Sarab (Northwestern of Iran), between August 2021 and November 2021. Participants were 18–65 years old referring to the Sarab health centers. The sample size was calculated based on information derived from a similar study (18) and a confidence level of %95, Z=1.96, SD=1.1, Mean=19.9, 379 samples (184 men, 195 women).

The sampling method was multi-state cluster random. The city of Sarab includes 4 health centers, each of which was considered as a cluster, then the participants who had inclusion criteria were randomly selected from these centers by health records. After explaining the study aims, informed consent was obtained from them. To complete the questionnaires were applied face to face interviews. Interviews were performed on the doorstep and lasted almost 25 minutes.

Inclusion criteria were having 18–65 years old and consent to participate in the study. The exclusion criteria did not have psychotic disorder and reluctance to participate in the study.

Study tools and scoring. Data were collected by demographic information questionnaire, Health Literacy for Iranian Adults (HELIA) and Health Promoting Lifestyle Profile II (HPLP-II).

**Demographic information questionnaire**: Demographic information includes participants' gender, age, marital status, job status, education level, and status of obtaining information related to health and disease.

Health Literacy for Iranian Adults (HELIA): We used the validated Health Literacy for Iranian Adults (HELIA) (19). This questionnaire consisted of 47 items and five dimensions: 1) Reading health information (4 items) measured using a five-interval Likert scale, ranging from 1 (completely difficult) to 5 (completely easy). The total score ranged from 4 to 20. The higher scores represented a high level of reading health information, 2) Understanding health information (7 items) rated on a 5-point scale ranging from 1 (completely difficult) to 5 (completely easy). The scores for understanding items ranged from 7 to 35. The higher scores determined the better condition for understanding. 3) Appraisal of health information (4 items), rated on a 5-point scale ranging from 1 (never) to 5 (always). The total scores for this dimension ranged from 4 to 20. The high level of scores indicated a high ability of appraisal of health information. 4) Ability to access health information (6 items) was scored five-interval Likert scale (always=5, most of the time=4, sometimes=3, seldom=2 and never=1). The scores were from 6 to 30 and a higher score showed a better ability to access health information. 5) Decision making (12 items) was measured five-interval Likert scale (always=5, most of the time=4, sometimes=3, seldom=2 and never =1). The scores for decision-making items ranged from 12 to 60. The higher scores were recoded to show a better condition. Cronbach's alpha for all of the dimensions of the questionnaire was measured > 0.7 (0.72–0.89) (19).

Health Promoting Lifestyle Profile II (HPLP-II): Health promotion behaviors were assessed using the standardized Persian version of the Health Promoting Lifestyle Profile II (20). This questionnaire has 52 items and 6 subscales: health responsibility (9 items; α=0.77), exercise (9 items; α=0.81), nutrition (9 items; α=0.74), self-actualization (9 items; α=0.73), interpersonal communications (9 items; α=0.72), and stress management (9 items; α=0.75). Each question in subscales was assigned a value of a 4-interval Likert scale (always=4, most of the time=3, sometimes=2, and never =1). The total score ranges from 52 to 208; a high score indicates the participants' greater tendency to adopt healthier behavior. The Cronbach's α for all questionnaires was measured 0.92 (20).

**Data analysis**: The participants' demographic information level of HL and health promotion behaviors were analyzed by the standard deviation (SD) and frequency (percentages), depending on data distribution.

The quantitative variables were analyzed using bivariate analyses (i.e., the independent samples T-test). Pearson's correlation coefficient was used to measure the relationship between HL and Health Promoting Lifestyle Profile II (HPLP-II). We used hierarchical regression analysis for HPLP-II in 2 steps. The demographic characteristics, including gender, age, marital status, job status, and education level, were entered in the first step. In the second step, along with the demographic characteristics, HL subscales were involved and was assessed R2 change. All statistical tests were analyzed using SPSSv.21 at significance level P <0.05.

**Ethical clearance**: This study was approved by the Sarab faculty of medical sciences (Number: IR.SARAB.REC.1399.002). Informed consent was obtained from the participants. Also, the questionnaires were anonymous.

## Results

Table 1 shows the demographic information of the participants. The average age of the participants was 35.26 ± 11.51. The majority of the participants (51.1 percent, n= 195) were females and had diploma or less than diploma qualifications (39.6%, n= 150). Approximately, 49% of the participants (n= 185) were unemployed. HPBs discovered no statistically significant differences in demographic factors. Table 1 shows that age (p-value= 0.006), gender (p-value= 0.012), and education level (p-value=0.001) all have statistically significant associations with HL.

**Table 1:** Relationship between HL, HPBs and some of demographic characteristics among adults

Variables		N (%)	HL Me ± SD	P-value	HPBs Me ± SD	P-value
**Age** *	18 to 27	116 (30.6)	136.12 ± 17.69	0.006	151.12 ± 21.62	0.720
	28 to 37	110 (29.0)	135.01 ± 19.30		148.73 ± 24.93	
	38 to 47	89 (23.5)	133.38 ± 17.55		148.92 ± 26.41	
	48 and higher	64 (16.9)	125.69 ± 25.54		146.92 ± 24.66	
**Gender** **	Male	184 (48.5)	130.78 ± 19.52	0.012	147.48 ± 24.65	0.181
	Female	195 (51.5)	135.89 ± 19.94		150.82 ± 23.78	
**Education level** *	Diploma & under diploma	150 (39.6)	128 ± 20.50	< 0.001	146 ± 23.83	0.345
	Duper diploma	47 (12.4)	133.85 ± 17.27		149 ± 22.09	
	Bachelor	135 (35.6)	138.89 ±17.07		151 ± 24.90	
	Masters & higher	47 (12.6)	134.34 ± 23.58		148 ± 25.49	
**Occupation** **	Employed	194 (51.2)	134.01 ± 19.87	0.903	149.79 ± 25.56	0.430
	Unemployed	185 (48.8)	133.72 ± 19.67		147.54 ± 22.70	
**Income (month)** *	2 m	148 (39.1)	132.40 ± 19.44	0.696	147.16 ± 21.22	0.232
	3–5 m	110 (29.0)	133.65 ± 19.87		148.67 ± 24.37	
	6	121 (31.9)	134.45 ± 20.51		152.20 ± 27.30	
**Marriage** **	Single	135 (35.6)	133.82 ± 20.54	0.766	148.48 ± 24.02	0.670
	Married	244 (64.4)	133.19 ± 19.54		149.60 ± 24.39	

* P-value based one-way ANOVA test

** P-value based t-independent test

Bivariate correlations between HL dimensions and HPBs are seen in Table 2. Using the Pearson correlation coefficient test, we discovered that HPBs exhibited statistically significant associations with all HL dimensions (p-value 0.05).

**Table 2:** Bivariate correlation matrix of the relationship between HL dimension and HPBs

Variables	1	2	3	4	5	6	Me ± SD
1=Reading health information	1						15.82 ± 3.80
2=Ability to access health information	0.696**	1					24.98 ± 4.98
3=Understanding health information	0.701 **	0.672**	1				29.82 ± 4.87
4=Appraisal of health information	0.452**	0.466**	0.656**	1			16.33 ± 3.64
5=Decision-making	0.338**	0.355**	0.505**	0.481**	1		46.94 ± 8.27
6= HPBs	0.353**	0.396**	0.444**	0.423**	0.680**	1	149.20 ± 24.23

* Correlation is significant at the 0.05 level (two-tailed)

To predict HPBs, we used hierarchical multiple linear regression. In step 1, demographic variables were not significant predictors of HPBs (p-value > 0.05, R2 total= 0.015), as shown in table 3. Demographic characteristics explained 1.5 percent of the variation in HPBs (F= 1.09; p-value=0.362), implying that demographic variables account for nearly 1.5 percent of the variation in HPBs. Step 2 included the addition of the HL dimensions, which explained an additional 48.3% of the variation (F= 70.23; p-value 0.001). In total, demographic factors and HL dimensions were able to account for around half of the variation in HPBs.

**Table 3:** Hierarchical linear regression for prediction HPBs through demographic characteristics and HL

Variables	β	R2 change	F change	SE	P-value
Step 1					
Age	0.078	0.015	1.097	0.134	0.362
Gender	0.066			2.69	
Job	0.035			3.063	
Marriage	0.057			3.13	
Education level	0.051			1.26	
Step 2					
Age	0.040	0.483	70.236	0.10	< 0.001
Gender	0.010			1.95	
Job	0.061			2.24	
Marriage	0.019			2.28	
Education level	0.041			0.93	
Reading health information	0.048			0.37	
Ability to access health information	0.115*			0.28	
Understanding health information	0.141*			0.31	
Appraisal of health information	0.062			0.33	
Decision-making	0.606*			0.13	
Total R2	-	0.497	-	-	-
Adjusted R2	-	0.483	-	-	-

* P < 0.05

## Discussion

HL is an important factor in the efficiency and effectiveness of health education and promotion programs that lead to adopting HPBs. There was a relationship between low level of HL and poor HPBs level.

We found that HL level was associated with some demographic characteristics, including age, gender and education level. Our findings were consistent with previous studies in Iran (3, 21–23), Ghana (8), Turkey (4), Polish (9) and Taiwan (11). In this study, individuals tended to be old have low levels of education, then they are more likely lower mean HL scores and are more likely to indicate poor HPBs levels. Levin-Zamir et al. (2016) revealed that individuals with 12 years of education have better HL (24). A study conducted by Aldosokey et al. (2021) in Egypt demonstrated that with increasing age in the elderly adults, the level of HL decreased due to a decrease in their cognitive ability and comprehension and remembering (15). Health is more important for women than men, and health-related information-seeking is better in women than men (25). Therefore, HL in women is better compared to men. However, in the gender variable and its relationship with HL have been reported different results in studies (24, 26–29). This difference can be due to cultural diversity and differences in the target group in different studies.

In the present study, HL dimensions were significantly correlated with HPBs and the HL predicted 48.3% of the variation in HPBs. Decision-making was the strongest HL dimension in predicting of HPBs. This indicated the level of HL in adults affected by their HPBs. This finding is consistent with the other studies that showed a significant positive relationship between HL and health behaviors in adults aged 18–64 years (22, 23, 29), older adults (30, 31), and adults at risk for diabetes (12), patients with type 2 diabetes (28, 32), nursing students (33) and students (34). A systematic revive (2018) conducted by Fleary et al. demonstrated thirteen studies reported significant, linear relationships between functional and media HL and health behaviors in adolescents (35). A study was conducted in South Korea, which examined the association between e-health literacy and HPBs; overall model determined 46% of the total variance in HPBs (33). Another study among adults with type 2 diabetes in Iran revealed that HL dimensions explained 47.5% of the total variation in health-related quality of life (36).

Better HL accounts for more health-related information-seeking behavior in adults (25). A higher level of HL and health promoting behavior in older adults can lead to the higher quality of life (30). The study of the Levin-Zamir et al. indicated that lower HL leads to lower physical activity, higher body mass index and lower sun protection (24). A research among multiethnic groups of women in Taiwan revealed that HL explains a small portion of the variance in HPBs (6 percent of the variance) (11). In our study, adults aged 18–65 years were addressed, but this research only assessed women, especially multiethnic groups. To improve HPBs, it is important to identify the research gap in factors related to HPBs and HL, because, determining these factors can help to design appropriate and applied intervention programs.

Most of the health-related information is provided with written forms (15) in health care centers, such as pamphlet, booklet and checklist which needs a high level of reading, access, understanding, appraisal and decision making. In this study, the lowest mean score of HL was in the age group of 48 years and upper than. Also, the decision-making dimension was the most critical determinant of HPBs in recent research. This dimension requires a high level of education. The findings of a study by Buck and Frosini showed that people who have no or low level of education are 5 times more likely to decide to adopt unhealthy behaviors (37). Thus, to improve HL and subsequent HPBs, it is essential to design and use appropriate educational materials for people with different literacy levels. It is recommended to perform further studies to find alternative HPBs predictors in adults because the HL and demographic variables account for 49.7 percent of the variation in HPBs variation.

There was some limitation in the present research. Due to the nature of cross-sectional studies, the results of this study cannot be causal. This study was conducted in Sarab city, which is situated in Northwestern of Iran. The language of this city people is Turkish and they have a different culture than the other province of Iran with Persian or the other languages. So, generalizing results should probably be applied to other provinces and ethnicities. As well, to reduce the bias in understanding and to evaluate questionnaires information, data were collected in the interview formats and doorstep. This can be strength of the present study.

In total, our research demonstrated that HPBs are positively associated with HL. In addition, decision-making was the strongest HL dimension in predicting of HPBs. Therefore, designing programs to promote HL and HPBs should be provided in adults with focus on decision-making dimension, aging groups, gender and education level.
